# Biometrics: Accessibility challenge or opportunity?

**DOI:** 10.1371/journal.pone.0194111

**Published:** 2018-03-22

**Authors:** Ramon Blanco-Gonzalo, Chiara Lunerti, Raul Sanchez-Reillo, Richard Michael Guest

**Affiliations:** 1 University Group for Identification Technologies, Department of Electronic Technology, University Carlos III of Madrid, Leganés, Madrid; 2 School of Engineering and Digital Arts, University of Kent, Canterbury, United Kingdom; National Institute of Technology Rourkela, INDIA

## Abstract

Biometric recognition is currently implemented in several authentication contexts, most recently in mobile devices where it is expected to complement or even replace traditional authentication modalities such as PIN (Personal Identification Number) or passwords. The assumed convenience characteristics of biometrics are transparency, reliability and ease-of-use, however, the question of whether biometric recognition is as intuitive and straightforward to use is open to debate. Can biometric systems make some tasks easier for people with accessibility concerns? To investigate this question, an accessibility evaluation of a mobile app was conducted where test subjects withdraw money from a fictitious ATM (Automated Teller Machine) scenario. The biometric authentication mechanisms used include face, voice, and fingerprint. Furthermore, we employed traditional modalities of PIN and pattern in order to check if biometric recognition is indeed a real improvement. The trial test subjects within this work were people with real-life accessibility concerns. A group of people without accessibility concerns also participated, providing a baseline performance. Experimental results are presented concerning performance, HCI (Human-Computer Interaction) and accessibility, grouped according to category of accessibility concern. Our results reveal links between individual modalities and user category establishing guidelines for future accessible biometric products.

## Introduction

Biometrics have an important role in mobile security systems. They are reliable and convenient to use, providing quick authentication without the need to memorise a sequence, unlike passwords and passcodes. Biometrics can be applied to a variety of contexts. For example, users can authenticate themselves in a mobile banking application using a single modality (such as face or voice), or combination of different biometric modalities, to enable a secure payment directly from their smartphone.

Biometric systems are replacing conventional authentication mechanisms such as PIN or graphical ‘pattern’ password to perform functions such as unlocking the screen of the device. Furthermore, biometrics can be used in combination with conventional methods to enhance the security around the protection of important sensitive data [1]. The presentation of a biometric sample/characteristic to the device can be straightforward, sometimes even transparent without the user even noticing that the capture process is occurring—for example, performing face authentication by simply looking at a device.

### Accessibility as an opportunity

Ensuring that personal data is secure, it is of great importance that security systems are reliable and easy to use for as wide a cross-section of the population as possible. Ideally any system must not be inaccessible to groups such as the elderly, people with disabilities, or those with little knowledge of technology.

Given that mobile authentication methods are at a stage of entering implementational maturity, there is a great opportunity to inspire the deployment of new systems that have the desirable characteristics of universality, ease of use and high performance, with the potential to make daily tasks much easier for a wide population.

### Experiment background-CRMF

The experiment reported in this paper continues a mobile biometrics accessibility research line [2]–[4] involving the collaboration of the ‘Centre for the Recovery of Persons with Physical Disability of Madrid’ (CRMF) based in Madrid (Spain). The Centre offers a range of rehabilitation facilities for physical or mental disabilities. Previous experiments have evaluated the accessibility of mobile devices apps for authentication with handwritten signature and fingerprint recognition, the latter performed using external devices [2]. The results of these works were utilised as guidelines for future developments and applied to this experiment. In this present work, some of the most common authentication modalities in mobile devices were utilised. The biometrics modalities tested were speaker recognition, face and fingerprint (using the integrated smartphone sensor). Non-biometric modalities were PIN and pattern. The three main research objectives in this experiment were:

- To test the accessibility of the common biometric authentication modalities in mobile devices.
- To compare traditional authentication mechanisms with biometrics in terms of performance, HCI (Human Computer Interaction) and accessibility.
- To establish groups/clusters of test subjects with respect to accessibility concerns and establish links between these groups and modalities and/or preferences.

The experiment consisted of asking the test subjects to authenticate themselves on a mobile app in order to withdraw money from a fictitious ATM (represented by a tablet computer) scenario on which they had previously enrolled. Authentication was performed through the modalities described above (biometric and non-biometric). Once the test subject completed the authentication in all modalities, the fictitious ATM shows a fake note of €20 on the screen. This is more a first approach to a realistic scenario than an operational environment. The reason to withdraw the money once the authentication is performed in all modalities is to gather feedback from all of them.

This paper is organised as follows: a brief state-of-the-art regarding accessibility in biometrics is presented in the next section, followed by a description of the experimental design and methodology used. Results are reported within the results section and finally conclusions and best practices are discussed.

## State of the art

Though there are several works on usability in biometrics, such as [5]–[7], there are only a few previous works in the area, as accessibility has not still gained too much attention in biometric recognition systems [8]. Previous studies highlight the main difficulties that people with accessibility concerns may face when interacting with biometrics [3]. A technical report within the ISO/IEC/JTC1/SC37 –Biometrics, namely ISO/IEC TR 29194:2015[9] contains best practices for biometric systems implementation with respect to different disabilities.

Further experiments have analysed the convenience of using specific modalities for elderly [2], [10] and for people with visual impairments [11]–[13], concluding that biometric recognition could ease common tasks such as banking transactions or mobile authentication. In studies of biometrics with elderly users, outcomes suggest that users have initial anxieties due to distrust and fear of unknown technologies. Recommendations reached during early accessibility experiments have been implemented in active banking apps, resulting in enhanced user experiences [14].

In [4] the authors carried out an accessibility evaluation of a banking app which utilised biometric authentication, implemented according to EN 301 549—Accessibility requirements suitable for public procurement of ICT products and services in Europe [15]. Participants ranked fingerprint and handwritten signature modalities highly in terms of comfort and security.

Accessibility is intrinsically linked to the term “usability”. There are several works in the literature regarding usability in biometrics and, most recently, concerning mobile devices [16]–[18]. Most of those studies are based on ISO 9241–11:1998 [19] and the NIST [20] definition of usability “the extent to which a product can be used by specified users to achieve specified goals with effectiveness, efficiency and satisfaction in a specified context of use”. Where effectiveness, efficiency and satisfaction are defined as:

- Effectiveness: “Accuracy and completeness with which users achieve specified goals”.
- Efficiency: “Resources expended in relation to the accuracy and completeness with which users achieve goals”.
- Satisfaction: “Freedom from discomfort, and positive attitudes towards the use of the product”

Some well-known investigations are the UK passport project conducted by Atos [21] and the HBSI framework developed by the Purdue University [22]. Both studies commenced following the NIST directives of usability and attempted to categorize the main usability measurements. Accessibility considerations have not, to date, been considered fully in the context of these frameworks.

## Evaluation set-up

This section contains the information related to the experimental evaluation, including test subjects’ characteristics, requirements, scenarios and methodology (based on the ISO/IEC 19795–2:2007 [23] and authors’ previous work [24]).

### Test subjects

A total of 41 test subjects took part in the experiment. 21 test subjects had accessibility concerns as determined by the CRMF. It is relevant to remark the difficulty of finding users with accessibility concerns and willing to participate in this kind of experiments. Current research experiments related to accessibility have similar number of participants [11], [25]. A total of 30 users interested in this experiment a priori, refused to participate due to lack of confidence in the technology. CRMF groups are explained in this section, within the physical and psychical disabilities. The demographic data of the test subjects is in Table 1.

**Table 1:** Demographic data of the test subjects (CRMF and Control test subjects).

	CRMF	Control
Gender	14 males / 7 females	10 males / 10 females
Age	12 (18–30) 5 (31–45) 4 (46–60)	6 (18–30) 8 (31–45) 4 (46–60) 2 (61+)
Academic degree	8 Univ. degree 5 High school 7 Primary studies 1 No studies	12 Univ. degree 5 High school 3 Primary studies
Experience	18 Mobile devices 10 Computers 2 Biometrics	20 Mobile devices 15 Computers 5 Biometrics
Groups	5 HAD*^1^ 11 LED*^2^ 2 VID*^3^ 17 CLD*^4^	

*^1^HAD: Hands/arms disabilities,

*^2^LED: legs disabilities,

*^3^VID: Visual disabilities,

*^4^CLD: Cognitive/learning difficulties

We considered subgroups of test subjects according to the physical or mental disabilities presented. There are many other existing accessibility concerns [9] but we only considered those present within the CRMF group of test subjects. CRMF test subjects were divided in accordance with their accessibility concerns (it is important to note that some test subjects may have more than one disability, being included in more than one group), namely:

#### Physical disabilities

- Hands/arms disabilities—HAD: Total or partial inability to use hands/arms properly when carrying out common tasks. In this experiment, this group will potentially have issues when interacting with the mobile device: handling it and/or touching the screen.
- Legs disabilities—LED: Total or partial inability to walk properly. Due to the equipment, this group rely on (e.g. wheelchairs or crutches), there could be different inconveniences in the interaction, such as difficulties to handle the mobile device with both hands.
- Visual disabilities—VID. Users who have difficulty in perceiving visual information (overall severe blindness).

#### Psychical disabilities

Cognitive or learning difficulties—CLD. Total or partial inability to understand instructions, memorize steps, talk properly or reading signs among others.

Control test subjects have no particular characteristics and have been selected randomly, covering the main representative groups in terms of age, gender and technology knowledge. This group acts as the baseline.

### Devices used

Test subjects interacted with an Android app running on a OnePlus 3T smartphone (size: 152.7 x 74.7 x 7.35 mm and 5.5” screen). This device was selected as it incorporates an embedded fingerprint sensor and a 16 MP frontal camera. It also satisfies the requirements determined by previous work: capacitive screen and easiness to use. The fictitious ATM was a Sony Xperia Tablet Z (size: 266 x 6.9 x 172 mm and 10,1” screen), connected to the smartphone app via Bluetooth.

### Test subjects’ guidance and training

The entire experimental procedure was explained to all test subjects before starting the trial (CLD group needed further explanations by nurses). Moreover, the application offered information during each process. In addition, reminders as text messages on the screen are shown in all the stages. The evaluation is designed to be completed with an operator guidance, but the test subject is free to complete the process autonomously when possible.

### Evaluation workflow

The experiment consisted of two sessions separated by a minimum of one week. At the beginning of the first session, test subjects were given information about the aim of the study and they were asked to sign a consent form to participate. They were required to complete a survey to collect demographics at the beginning of the session, where they were also asked about their opinion of biometrics and if they had any kind of experience with the technology. All gathered data have been handled according to the EU Data Protection Directive (Directive 95/46/EC) and the Spanish national data protection law (LOPD) [26] and all participants were properly informed about it. This specific study was reviewed and approved by the ethics committee of the University Carlos III of Madrid [27] before the study began. In order to start the evaluation, participants had to sign an agreement with this respect.

Participants were next requested to enrol each biometric and non-biometric characteristic in order: i) take 5 frontal images of their face; ii) enter a 4 digit PIN 3 times; iii) read out loud a sentence shown in the screen while pressing a virtual ‘sample’ button also on the screen and repeat the presentation 3 times (the same sentence was used during the whole evaluation). The sentence was in Spanish: “Mi voz es la clave que no tengo que recordar” (translation: “My voice is the key I do not have to remember”); iv) draw a pattern 3 times and finally, v) follow the instruction of the Android interface to enrol a single fingerprint on the system. The number of presentations per modality is the required number by each of the biometric algorithms applied.

After the enrolment, test subjects were presented with a scenario where they were required to withdraw money from a fictitious ATM. In order to do so, they needed to verify themselves on the Bluetooth-connected smartphone to conclude the transaction. During enrolment test subjects were supervised by an operator, ensuring that they had a clear idea of how to proceed in each step. During the verification task subjects were not given any detailed instructions apart from the indications provided by the app. During the verification task, subjects had to donate samples in the same order as enrolment, but providing only one sample of each modality.

**Fig 1:**
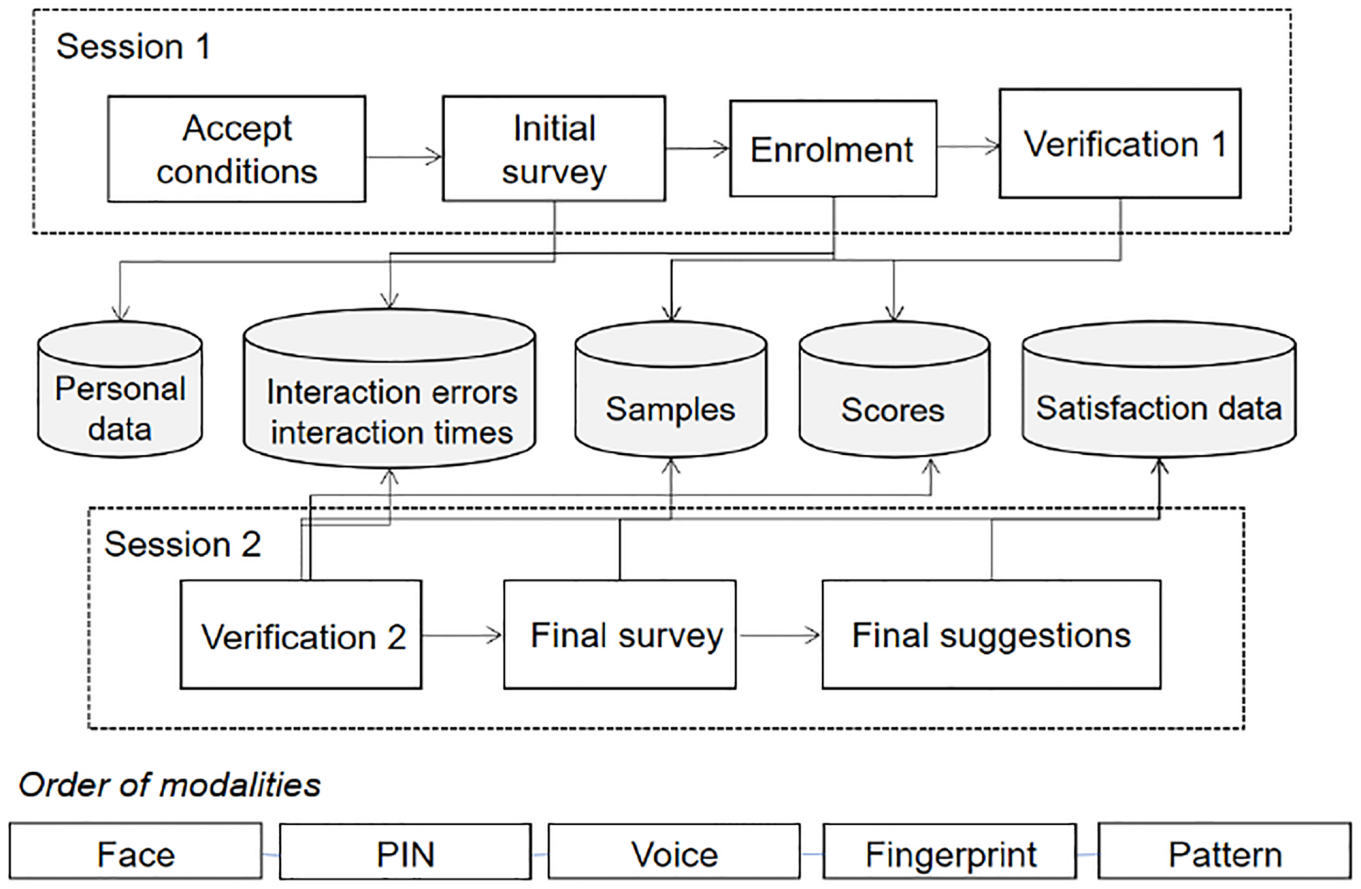
Evaluation map.

The second session took place a minimum of one week after the first session. Test subjects were presented with the same scenario that required the withdrawing of money from the ATM represented by the tablet using the mobile app. For the authentication in this second phase, subjects were asked to present face, PIN, voice, fingerprint and pattern. At the end of the session they had to complete a questionnaire concerning the overall experience they had with the app, the difficulties they may have encountered and their preference on the modalities. The experimental process is summarized in Fig 1.

### Biometric modalities characteristics

The speaker recognition system (the Kivox 360 tool [18]) was provided by Agnitio and returns several results from a biometric process regarding sample quality and performance. Nevertheless, not all the results the tool provides are within the scope of this paper. Therefore, for this experiment, a voice sample was sent to a server that returns a sample quality feature and biometric verification decision (yes/no). We have quantized the quality to sufficient or low quality.

Fingerprints were collected using the Android interface that is available in smartphones provided with fingerprint sensor. The Android operating system has a limit of 5 fingerprints that can be stored on a single device, so this restricted the experiment as it was not possible to use fingerprints in the second session (only 5 users can be registered at the same time). The fingerprint recognition system does not allow image extraction or percentage match result reporting, only returning a ‘yes/no’ decision.

**Fig 2:**
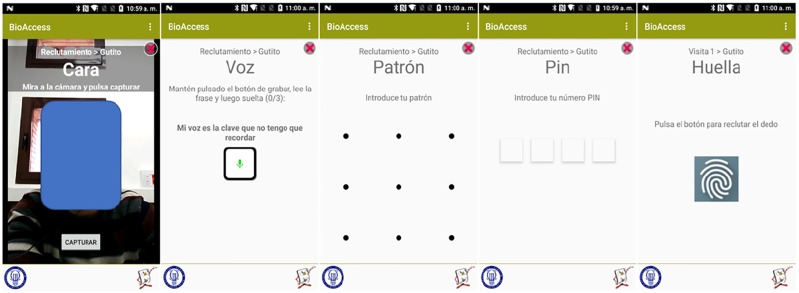
Examples of the app interfaces. From left to right (Spanish): Face, Voice, Pattern, PIN and Fingerprint.

No face recognition algorithms were actively applied during the evaluation process meaning that test subjects took “selfies” without any quality or face detection feedback. However, they were instructed to locate the face frontal and within the boundaries of a guiding bounding box. Once all data was collected, a Viola-Jones based face detection algorithm was utilised [28]. Images were cropped by the bounding boxes generated and used as an input to a SIFT based algorithm [29] in order to be compared to the reference images taken during the enrolment stage. SIFT is applied because it is resistant to occlusion, scale and orientation changes. SIFT represents a face image by many descriptors. To compare two images represented in their respective sets of SIFT descriptors, a Euclidean distance between these descriptors was calculated. If the distance is below a prefixed threshold, it is considered a match. The final matching score is computed as the number of paired descriptors divided by the number of available descriptors. The number of detected faces in the images was used as a sample quality parameter to check whether test subjects are able to properly take selfies suitable for face recognition. Sample images of the app interfaces are in Fig 2.

### Experimentation

Once the evaluation had finished, data was processed to derive performance, test subject interaction and accessibility results.

#### Performance results

In the non-biometric modalities (PIN and pattern), we simply compared the sample with the enrolment template and return “match” if the numbers are the same or “non-match” otherwise. In speaker recognition, our tool directly returned the quality result and the comparison between the verification sample and the enrolment template. As described, fingerprints were managed by Android returning a yes/no decision for matched comparisons. For face recognition, a threshold was established to calculate the verification errors and all the results under the threshold were considered as a non-match. The threshold was computed through the N:N comparisons of all images in the database, based on distance of samples in same class (FNMR—False Non-Match Rate) and between classes (FMR—False Match Rate). Nevertheless, as non-mated comparisons are not the objective of this work, only verification errors from comparisons where the template and samples are from the same test subject were performed. We calculate one comparison per verification in each modality, except for face recognition, where we have access to the images. Five comparisons per verification image were calculated (one per each image taken during enrolment), involving 10 comparisons per test subject in total.

#### HCI results

HCI evaluation, also known as usability evaluations, are widely utilised for ICT product development. Several ISO standards cover user-system interaction and provide means to assess usability, such as the “Software product Quality Requirements and Evaluation” (SQuaRE) standards [30]. Nevertheless, usability evaluation within biometric recognition systems brings specific challenges, which must be assessed. We have used the usability definition of ISO 9241:1998 [19] to derive HCI results. Therefore, metrics applied are effectiveness (incorrect interactions), efficiency (time taken to perform tasks) and satisfaction (fulfilment of test subject expectations).

#### Accessibility results

To provide categorisation to accessibility results, we have separated them as defined by test subject subgroups in the Evaluation set-up section. Moreover, we have included two factors indicating the extent to which the test subjects could complete the experiment and another factor related to acceptability:

- Number of test subjects who could not begin interaction with a modality. Test subjects with physical or psychical disabilities that are unable to perform any required actions of a modality transaction. Moreover, control test subjects (without accessibility concerns a priori) may find it difficult or impossible to use some modalities.
- Number of test subjects who could not complete the section. Test subjects who started a specific section but could not complete (e.g. test subject forgot the PIN or consumed all attempts in fingerprint recognition).
- Number of test subjects who did not want to start the section—due to distrust in the technology, fear of damage, nervousness or other reasons. This last factor could not be considered as an accessibility parameter, but more as a factor of acceptability and/or trust in the technology.

## Results

The results are divided in two groups: CRMF and Control, the latter providing a baseline performance. We have also split results according to different subgroups within the CRMF. This Section contains purely results. Interpretation of all results is within the conclusions.

### Performance results

This section contains the results of the verification comparisons from both biometric and non-biometric modalities. Sample quality results are provided for speaker and face recognition. Fingerprint recognition provided by the Android OS does not return sample quality results, thus they are not obtainable.

#### Speaker recognition

In speaker recognition, quality and performance results were returned. In Table 2, numbers of low quality voice samples during the sessions are shown by groups.

**Table 2:** Percentage of low quality voice samples aquired during the evaluation.

Test Subjects		Session		
		Enrolment	Visit 1	Visit 2
CRMF	HAD	0%	40%	0%
	LED	0%	18%	18%
	VID	0%	0%	0%
	CLD	0%	35,3%	5%
Control		0%	0%	0%

In Table 3 the verification errors are split in sessions and groups of test subjects. A verification error is produced when the similarity between the sample and the template is lower than the predefined threshold. Each test subject had to complete a single verification, using 3 attempts to achieve this. It is important to remark that test subjects who could not complete the speaker verification, alongside attempts with insufficient quality (as previously shown in Table 2) are not included.

**Table 3:** Percentage of speaker verification errors during the evaluation.

Test Subjects		Session	
		Visit 1	Visit 2
CRMF	HAD	0%	0%
	LED	0%	22%
	VID	0%	50%
	CLD	0%	0%
Control		0%	0%

#### Face recognition

Shown in Table 4 are the number of non-detected faces by the different groups during the evaluation.

**Table 4:** Percentage of non-detected faces during the evaluation.

Test Subjects		Session		
		Enrolment	Visit 1	Visit 2
CRMF	HAD	3,8%	16,67%	16%
	LED	3,5%	8,34%	0%
	VID	0%	0%	0%
	CLD	2,3%	0%	0%
Control		3%	2,91%	0%

In Table 5 are the verification errors split in sessions and groups of test subjects. It is important to remark that test subjects who could not complete the face verification, alongside attempts with insufficient quality (as shown in Table 4) are not included.

**Table 5:** Percentage of face verification errors during the evaluation.

Test Subjects		Session	
		Visit 1	Visit 2
CRMF	HAD	11,7%	25%
	LED	24%	41,8%
	VID	40%	80%
	CLD	11,76%	30,58%
Control		24%	25%

#### Fingerprint recognition

All test subjects who attempted the fingerprint enrolment completed the process. Table 6 describes the verification errors of fingerprint recognition in Visit 1.

**Table 6:** Percentage of fingerprint verification errors during the evaluation.

Test Subjects		Session
		Visit 1
CRMF	HAD	0%
	LED	9%
	VID	0%
	CLD	0%
Control		0%

#### Use of the PIN

Problems at this stage occurred when test subjects forgot their PIN even during the enrolment (when the repetition of a number on 3 occasions was required) and when they were not able to properly press the device screen (e.g. hands disabilities). Table 7 contains the number of errors within the PIN modality during the different phases of the experiment.

**Table 7:** Percentage of PIN input errors during the evaluation.

Test Subjects		Session		
		Enrolment	Visit 1	Visit 2
CRMF	HAD	6,6%	50%	42,8%
	LED	0%	0%	40%
	VID	0%	0%	75%
	CLD	1,9%	30%	48%
Control		0%	0%	53%

#### Use of the pattern

Authentication by the finger pattern returns a “yes/no” decision. Errors in this stage are caused by forgetting the previously drawn pattern and by not being able to link the grid points. Table 8 contains the number of errors within the pattern modality during the different phases of the experiment.

**Table 8:** Percentage of pattern input errors during the evaluation.

Test Subjects		Session		
		Enrolment	Visit 1	Visit 2
CRMF	HAD	17,6%	0%	20%
	LED	16,2%	0%	52,17%
	VID	0%	0%	100%
	CLD	17,54%	0%	32%
Control		7,93%	13,04%	39,4%

### HCI results

Metrics to obtain HCI results are outlined by the ISO 9241:1999 definition but adapted to the particularities of this experiment:

- Effectiveness. As a measure of the test subjects’ incorrect interactions, we have counted the number of times test subjects did not interact with the system as instructed for each modality.
- Efficiency. We have measured the overall interaction time for each modality.
- Satisfaction. Test subjects completed surveys at the beginning and at the end of the experiment. Moreover, the evaluation operator collected all suggestions and opinions during the experiment.

As above, all measurements are divided in the predefined test subject groups.

#### Effectiveness

There are several ways to perform an incorrect interaction in this experiment. In fact, users discovered many new ways of proceeding incorrectly during the evaluation. A thorough analysis would provide extensive work in itself—in that regard we have only accounted the number of incorrect interactions and cited the most common for each modality.

Incorrect interactions in speaker recognition occurred due to problems when reading the text, inconveniences when pressing the button to record, inconveniences when holding the mobile device or to nervousness when talking. Table 9 summarizes the incorrect interactions in speaker recognition.

**Table 9:** Percentage of incorrect interactions during speaker verification.

Test Subjects		Session		
		Enrolment	Visit 1	Visit 2
CRMF	HAD	6,2%	28,5%	0%
	LED	0%	28,5%	26,6%
	VID	0%	0%	60%
	CLD	0%	26,08%	5,5%
Control		0%	4,76%	13,04%

In the case of face recognition, incorrect interactions occurred when test subjects attempted to direct the camera onto a facial area, problems in holding the mobile device and when pressing the button to take the picture. Incorrect interactions are shown in Table 10.

**Table 10:** Percentage of incorrect interactions during face verification.

Test Subjects		Session		
		Enrolment	Visit 1	Visit 2
CRMF	HAD	3,8%	16,6%	16,6%
	LED	3,5%	0%	0%
	VID	0%	0%	0%
	CLD	2,29%	11,76%	0%
Control		0%	0%	0%

Test subject interaction problems during the fingerprint recognition were mainly related to subjects incorrectly placing the finger on the sensor and keeping their finger stationary during capture. Table 11 summarises incorrect interactions during the evaluation.

**Table 11:** Percentage of incorrect interactions during fingerprint verification.

Test Subjects		Session	
		Enrolment	Visit 1
CRMF	HAD	0%	0%
	LED	0%	0%
	VID	0%	0%
	CLD	1,92%	0%
Control		3,22%	4,76%

Effectiveness errors in the non-biometric modalities were related to forgetting the PIN/pattern and not being able to properly interact with the screen. Errors related to forgetting the PIN/pattern are already included in Tables 7 and 8. Errors related to the inability to properly interact with the screen are documented in the Accessibility results section as they are directly related to accessibility concerns.

#### Efficiency

This factor is related to the time spent performing a specific task. In this experiment, we have measured the time spent in each modality and session in order to assess the efficiency evolution between sessions (learnability) and compare between modalities. Time in enrolment includes an increased number of individual captures resulting in enrolment completion times that are always longer than verification times.

Time spent in speaker recognition starts with the test subject pressing the button to record the voice at first attempt and finishes as they release the button to finish the last recording (3 samples in enrolment and 1 in verification). Table 12 details the average times and standard deviations for each of the test subject groups and sessions.

**Table 12:** Average and standard deviation of times in seconds spent in speaker recognition.

Test Subjects		Session		
		Enrolment	Visit 1	Visit 2
CRMF	HAD	48,16 ± 9,23	16,05 ± 5,80	17,11 ± 2,72
	LED	43,25 ± 11,46	10,77 ± 3,10	18,2 ± 6,90
	VID	33,35 ± 12,76	7,5 ± 0,80	17,28 ± 1,7
	CLD	43,64 ± 9,70	13,70 ± 5,40	19,03 ± 10,52
Control		34,24 ± 3,45	8,32 ± 2,03	11,31 ± 2,70

Time spent in face verification starts when the test subject presses the capture button for the first time and ends when the test subject takes the last picture (5 samples during the enrolment and 1 during verification). Times for face verification are shown in Table 13.

**Table 13:** Average and standard deviation of times in seconds spent in face verification.

Test Subjects		Session		
		Enrolment	Visit 1	Visit 2
CRMF	HAD	24,30 ± 8,20	11,15 ± 3,71	10,05 ± 6,06
	LED	32,01 ± 8,70	9,97 ± 10,00	9,68 ± 4,06
	VID	31,71 ± 8,46	5,69 ± 1,78	8,36 ± 0,24
	CLD	29,03 ± 7,09	8,72 ± 4,38	11,44 ± 6,29
Control		28,14 ± 9,76	4,74 ± 1,83	6,49 ± 2,67

Fingerprint recognition starts when the test subject presses the sensor for the first time and ends when the test subject releases the sensor for the last time (18–22 samples during the enrolment and 1 during verification). Timing results for fingerprint verification are shown in Table 14.

**Table 14:** Average and standard deviation of times in seconds spent in fingerprint recognition.

Test Subjects		Session	
		Enrolment	Visit 1
CRMF	HAD	72,58 ± 18,27	9,71 ± 5,18
	LED	59,38 ± 16,93	8,21 ± 4,58
	VID	52,61 ± 6,46	9,14 ± 0,91
	CLD	72,15 ± 21,39	8,58 ± 4,45
Control		64,39 ± 21,16	4,95 ± 2,53

The use of PIN starts when the test subject presses the first digit and ends when the test subject presses the last digit (3 times during the enrolment and 1 during verification). Time results for the use of PIN are shown in Table 15.

**Table 15:** Average and standard deviation of times in seconds spent in the use of PIN.

Test Subjects		Session		
		Enrolment	Visit 1	Visit 2
CRMF	HAD	25,42 ± 16,01	10,67 ± 4,02	11,25 ± 5,04
	LED	19,68 ± 12,57	8,15 ± 3,47	9,59 ± 6,45
	VID	15,61 ± 3,30	6,39 ± 0,67	7,23 ± 1,03
	CLD	19,52 ± 11,3	8,03 ± 3,39	10,00 ± 5,56
Control		10,46 ± 5,23	3,84 ± 1,01	5,19 ± 2,24

The use of pattern starts when the test subject presses the first point of the grid and ends when the test subject presses the last point of the pattern (3 times during the enrolment and 1 during verification). Time results for the use of pattern are shown in Table 16.

**Table 16:** Average and standard deviation of times in seconds spent in the use of pattern.

Test Subjects		Session		
		Enrolment	Visit 1	Visit 2
CRMF	HAD	14,28 ± 7,28	6,35 ± 2,59	7,58 ± 4,86
	LED	11,15 ± 6,24	4,66 ± 2,27	12,46 ± 9,01
	VID	10,48 ± 4,67	4,74 ± 1,09	-
	CLD	13,04 ± 5,87	5,63 ± 2,36	8,25 ± 5,14
Control		7,27 ± 5,14	3,10 ± 1,16	6,03 ± 4,41

Absence of times mean no test subject within a specific group could finish the session.

#### Satisfaction

**Fig 3:**
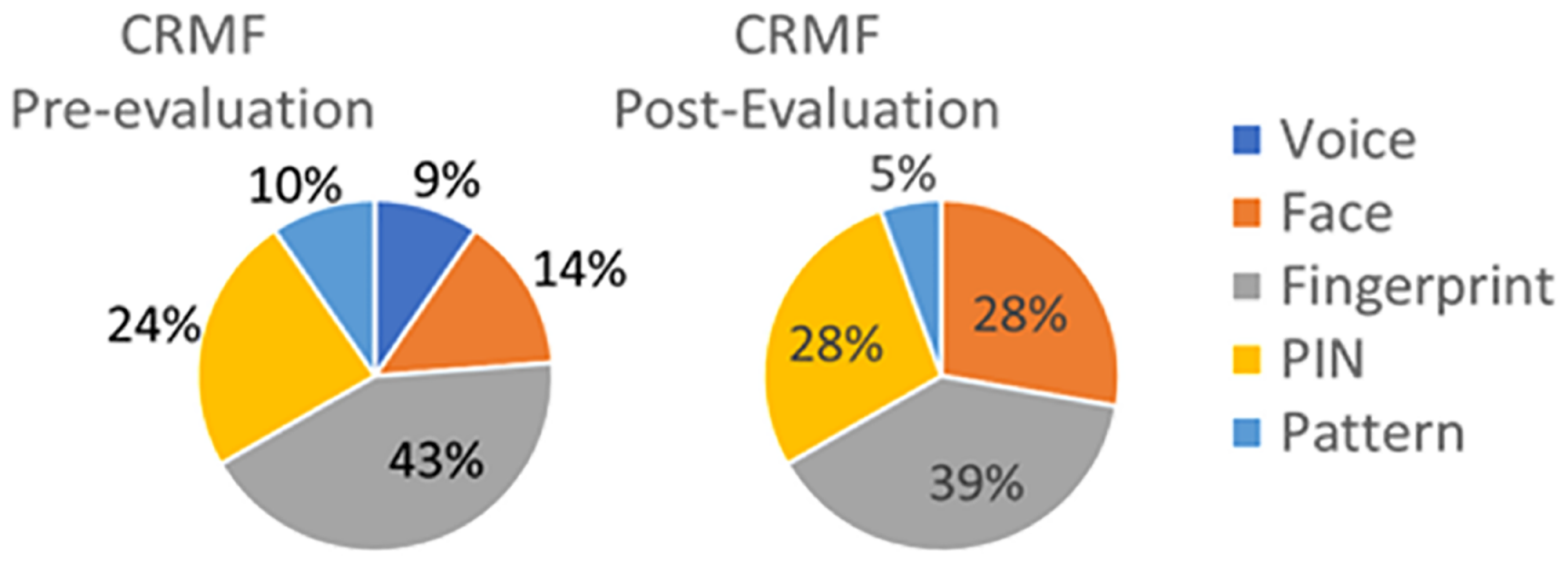
Results from survey question from CRMF test subjects regarding modality preferences before and after the experiment.

**Fig 4:**
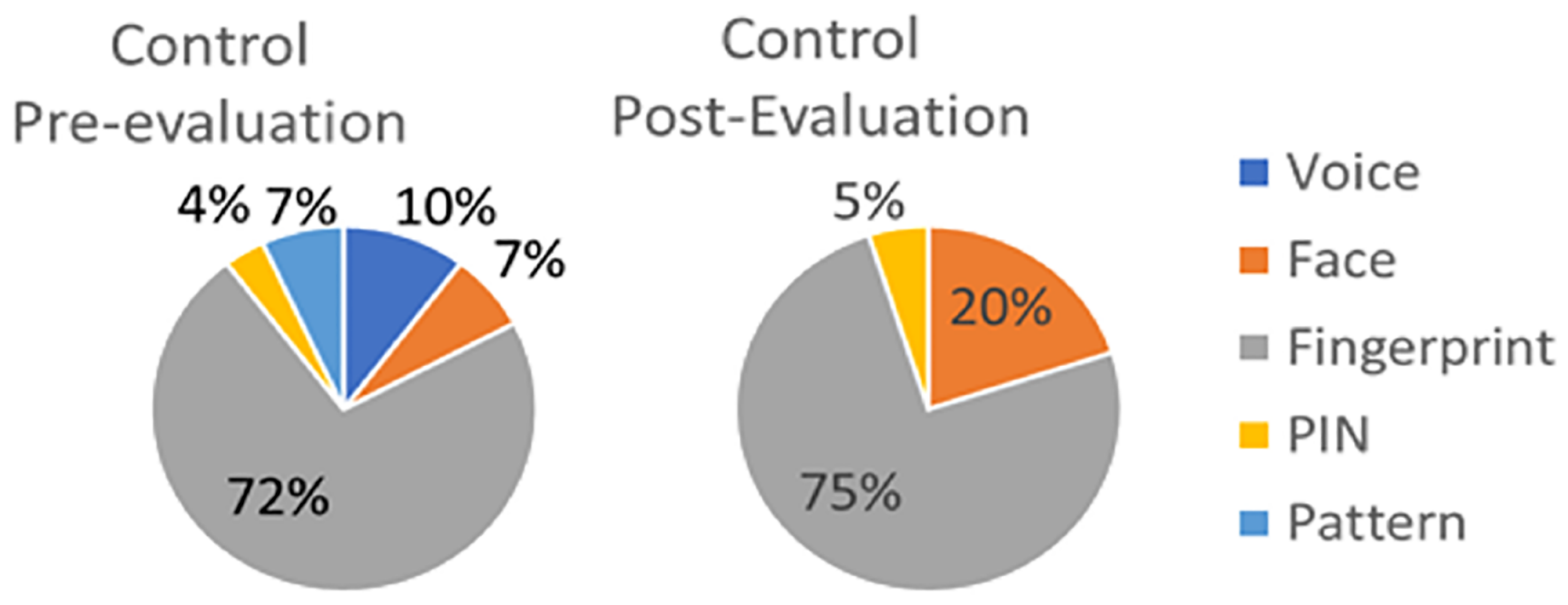
Results from survey question from Control test subjects regarding modality preferences before and after the experiment.

Test subject satisfaction was measured through pre- and post-evaluation surveys, test subjects’ suggestions and opinions compiled during the evaluation. Survey questions were related to demographics (results already included in the Evaluation set-up section) and to preferences about modalities and biometric recognition. Questions about preferences were the following:

- What would you prefer to use? Fingerprint/Face/Voice/PIN/ Pattern. Results to this question are in Fig 3 (CRMF) and Fig 4 (Control).
- Would you use biometric recognition to unlock your smartphone or PC? Yes/No, it is slow/No, it is uncomfortable/No, it is unsecure/No, it is difficult to use/I do not know/ Yes, but contactless.

All CRMF test subjects except one (citing security reasons) claimed before the experiment that they would use biometric recognition for locking their smartphone/PC. After the experiment, two users would not use biometrics, again citing security reasons, whilst another cited lack of comfort.

All Control test subjects would use biometric recognition for lock their smartphone/PC before the evaluation. Only one user changed their mind, preferring PIN at the end of the experiment.

- Would you use biometric recognition for making banking operations? Yes/No, it is slow/No, it is uncomfortable/No, it is unsecure/No, it is difficult to use/I do not know/ Yes, but contactless.

For the CRMF test subjects, before the experiment, 54% would use biometric recognition for banking transactions, 41% would not use it citing security reasons and 5% do not know the technology sufficiently. After the experiment, 70% would use biometrics for banking transactions and 30% would not use it due to security reasons. 72% of the Control test subjects before the experiment would use biometric recognition for banking transactions, 24% would not use it citing security reasons and 4% claimed lack of comfort. After the experiment, 86% would use biometrics for banking transactions and 14% would not use it due to distrust in its security.

### Accessibility results

This section contains the accessibility results in accordance with the defined metrics in the Evaluation set-up section.

- Number of test subjects who could not start the section. In Table 17 are the number of test subjects unable to start the different sections, divided into subgroups.
- Number of test subjects who could not complete the section. Table 18 contains the number of test subjects who could not complete some of the sections.
- Number of test subjects who did not want to start the section. Table 19 show the test subjects who did not want to use a particular modality.

**Table 17:** Number of test subjects who cannot use the different modalities.

Test Subjects		Modality				
		Voice	Face	Fingerprint	PIN	Pattern
CRMF	HAD	0	2	0	0	0
	LED	0	0	0	0	0
	VID	0	0	0	0	0
	CLD	1	2	0	0	2
Control		0	0	0	0	1

**Table 18:** Number of test subjects who could not finish the different modalities.

Test Subjects		Modality				
		Voice	Face	Fingerprint	PIN	Pattern
CRMF	HAD	0	0	0	1	2
	LED	0	0	0	2	6
	VID	0	0	0	1	2
	CLD	1	0	0	4	4
Control		0	0	0	5	4

**Table 19:** Number of test subjects who did not want to start the different modalities.

Test Subjects		Modality				
		Voice	Face	Fingerprint	PIN	Pattern
CRMF	HAD	0	1	0	0	0
	LED	0	0	1	0	0
	VID	0	0	0	0	0
	CLD	1	2	2	0	1
Control		0	0	0	0	0

## Conclusions and best practices

This work has allowed us to derive several conclusions and best practices that may be applied in further experiments and biometric system designs. These conclusions are split in performance, HCI and accessibility in accordance with the results obtained. Prior to derive conclusions, it is important to remind the sample size (not as big as in other usability studies), which is understandable for this type of research, pointing out that results cannot be trusted to a very high degree, but should be interpreted as a first approach.

### Performance

Regarding the sample quality acquired in speaker and face recognition, there is a remarkable difference between the Control group (almost no error) and CRMF test subjects. This is more noticeable in speaker recognition, where test subjects pressed a button while speaking leading to multiple difficulties for the HAD and CLD groups. In fact, most test subjects in these groups complained about the use of the button. Almost all groups had difficulties in both handling the device and framing the face for the face recognition task, especially for test subjects in the HAD group. Low quality face samples in Control test subjects occurred mostly due to bad lighting conditions.

Voice and fingerprint systems resulted in only a few verification errors. This fact could encourage the use of the fingerprint in smartphones, especially in Spain, where the national ID card includes fingerprints. On the other hand, face verification offered very poor results, caused by the difficulty of taking “selfies” for some groups and environmental conditions (e.g. bad lightning, reflections or non-uniform background). It is also important to note the amount of verification errors in the second session of PIN and pattern modalities. This occurred as the second session took place at least one week after the enrolment causing many test subjects to forget their credentials.

### HCI

HCI results are split in effectiveness, efficiency and satisfaction. Though these three terms are intrinsically related, we analyse results separately due to their different nuances.

#### Effectiveness

This factor was analysed only for biometric modalities as PIN and pattern effectiveness is included in performance results. Speaker verification is the modality that resulted in the most interaction errors. According to operator’s notes and users’ testimonies, most errors are caused by the recording button which was required to be pushed to record the biometric sample. Incorrect interaction with respect to this process did not lead to low quality samples, as the system did not detect the sample in the first instance and therefore, they were not processed. In the case of face verification, incorrect interactions led to low quality samples as shown in Table 4. Regarding fingerprint verification, operator’s notes describe some incorrect fingerprint presentations (e.g. rotations), but even in cases where test subject did not place the finger properly on the sensor, they were verified correctly.

#### Efficiency

Almost all modalities show high standard deviations in the execution time, meaning that not all test subjects have problems when interacting with the biometric system (even for test subjects with similar characteristics, i.e. in the same subgroup). In the efficiency results, a learning curve is noticeable in all modalities: test subjects learn how to use the system during enrolment (resulting longer execution times), they already have acquired practice when at the start of verification 1 (shorter times) and they lost ability one week after when verification 2 starts (medium-long times), including control group.

Test subjects commented about the large amount of time taken for fingerprint enrolment due to Android system requiring an average of 18–22 fingerprint samples in different positions to create a proper template. PIN and pattern resulted in shorter times in enrolment but with higher variability. Nevertheless, face and fingerprint verification return similar times during verification 2.

Test subjects within HAD subgroup had longer execution times across most modalities due to problems when handling the device for the first time. Subjects in the CLD subgroup showed significant differences in execution time between verification 1 and verification 2, due in most cases to memory loss, according to operators’ notes.

#### Satisfaction

CRMF test subjects showed their preference for modalities requiring less interaction with face verification considered fashionable according to users’ testimonies. Nevertheless, at the end of the evaluation, 28% of CRMF test subjects still prefer the use of PIN, considered it as safe and more familiar than biometrics. CLD test subjects showed their general enthusiasm for the use of biometric systems as it does not require codes or patterns to be memorised.

Control subjects prefer the use of fingerprint (75% at the end of the experiment) over face (20%) and PIN (5%). This fact reflects the increasing use of fingerprint verification in mobile devices, considered by end-users as more convenient and straightforward than traditional modalities (such as PIN or passwords).

Most of the test-subjects across all groups would use biometrics for guaranteeing the security of smartphones and PCs, but not for bank transactions. Before the experiment, 41% of CRMF test subjects and 24% of Control subjects claimed security reasons for not using biometrics for banking. This can be attributed to a fear of trust in new technologies which are generally unknown for most of the test subjects. At the end of the experiment, the confidence on the use of biometrics of some users increased: 70% of CRMF users and 14% of non-CRMF users would trust in biometrics for banking transactions.

### Accessibility

Test subjects who were not able to use a particular modality are those who require higher care and are not able to perform common daily actions by themselves. A single Control subject, aged between 60–81 years, could not understand how to proceed with the pattern modality.

Many subjects could not complete the second PIN and pattern verifications, because they had forgotten their credentials (even when they were encouraged to use familiar numbers and patterns). This fact reflects the usefulness of biometric recognition, not only for CRMF subjects but for everyone (some Control subjects had also problems remembering the credentials or directly forgot them).

Some CRMF subjects decided not to complete one or more modalities citing security concerns, fear of damage and privacy invasion. This concern mostly occurred with face and fingerprint verification.

### Best practices

As a summary of observations and recommendations, we derive a series of best practices for future designers and developers in order to motivate better biometric systems in terms of accessibility and universality.

- People with accessibility concerns feel nervous and anxious using new technologies. Special attention and care should be taken to make them feel calm during experiments
- Speaker verification systems are highly appreciated as a non-intrusive modality. Nevertheless, the inclusion of an extra element to record the voice (a button) could lead to rejections. Increasing automation may result in better user acceptance.
  - Other aspect to improve in speaker verification is the sentence to read. Many subjects have problems reading from screens (e.g. small letters, difficulties to read properly because hand tremor, etc.). One possible solution could be to repeat an audibly sentence previously played by the system.
- Introducing modalities which require high user interaction, may lead to confusion and rejections. In this case, several users disliked the use of the pattern.
- Authentication solutions may be adapted to each individual subject. Even though subgroups have been assessed in this work according to their characteristics, we found high variability among them, pointing out the requirement for more individualised solutions.
- Some subjects have more than one accessibility concern. This leads to situations where categorising users is unfeasible and add support to the previous recommendation.
- The fewer interactions with the system, the better. Transparent solutions involve fewer possibilities of incorrect interactions and reduce usage time. However, this fact could negatively affect system performance.
- The Android fingerprint enrolment procedure shows a fingerprint on the screen, that many subjects confused with the real fingerprint sensor and touched it several times. This led to longer times in the enrolment process, which was already considerable.
