# Correction: Biometrics: Accessibility challenge or opportunity?

**DOI:** 10.1371/journal.pone.0196372

**Published:** 2018-04-19

**Authors:** Ramon Blanco-Gonzalo, Chiara Lunerti, Raul Sanchez-Reillo, Richard Guest

The fourth author’s name is incorrect. The correct name is: Richard Guest. The correct citation is: Blanco-Gonzalo R, Lunerti C, Sanchez-Reillo R, Guest R (2018) Biometrics: Accessibility challenge or opportunity? PLoS ONE 13(3): e0194111. https://doi.org/10.1371/journal.pone.0194111
